# Early endothelial dysfunction in cholesterol-fed rabbits: a non-invasive *in vivo* ultrasound study.

**DOI:** 10.1186/1476-7120-2-10

**Published:** 2004-07-21

**Authors:** Marie-Claude Drolet, Éric Plante, Bruno Battistini, Jacques Couet, Marie Arsenault

**Affiliations:** 1 Groupe de recherche en valvulopathies, Institut de cardiologie de Québec, Centre de recherche Hôpital Laval, Université Laval, 2725 Chemin Sainte-Foy, Sainte-Foy (Québec) G1V 4G5 Canada

**Keywords:** endothelial function, atherosclerosis, cholesterol-fed rabbits, ultrasound.

## Abstract

**Background:**

Endothelial function in hypercholesterolemic rabbits is usually evaluated *ex vivo* on isolated aortic rings. *In vivo* evaluation requires invasive imaging procedures that cannot be repeated serially.

**Aim:**

We evaluated a non-invasive ultrasound technique to assess early endothelial function in rabbits and compare data with *ex vivo* measurements.

**Methods:**

Twenty-four rabbits (fed with a cholesterol diet (0.5%) for 2 to 8 weeks) were given progressive infusions of acetylcholine (0.05–0.5 μg/kg/min) and their endothelial function was assessed *in vivo* by transcutaneous vascular ultrasound of the abdominal aorta. Ex vivo endothelial function was evaluated on isolated aortic rings and compared to *in vivo* data.

**Results:**

Significant endothelial dysfunction was demonstrated in hypercholesterolemic animals as early as 2 weeks after beginning the cholesterol diet (aortic cross-sectional area variation: -2.9% vs. +4% for controls, p < 0.05). Unexpectedly, response to acetylcholine at 8 weeks was more variable. Endothelial function improved in 5 rabbits while 2 rabbits regained a normal endothelial function. These data corroborated well with *ex vivo* results.

**Conclusion:**

Endothelial function can be evaluated non-invasively *in vivo* by transcutaneous vascular ultrasound of the abdominal aorta in the rabbit and results correlate well with *ex vivo* data.

## Background

Endothelial dysfunction occurs early in the development of atherosclerosis. Historically, evaluation of endothelial function in small animals has been performed on isolated vessel segments, or vessels exposed by surgical procedures. Very few attempts were made to develop a method of analysis of endothelium-dependent relaxation *in vivo*[1-5]. In those studies, an invasive intravascular ultrasound approach was used. Correlation between results obtained *in vivo* and data on isolated arteries *ex vivo* was never assessed.

Non-invasive methods to study endothelial function in humans (e.g. ultrasound of the brachial artery) have been used for many years and have yielded an important amount of data [6-12]. Unfortunately this non-invasive technique has never been transposed to animal studies.

The objective of the current study was to assess the reliability of transcutaneous vascular ultrasound in order to evaluate endothelial function *in vivo* in rabbits and to compare this non-invasive method with results obtained *ex vivo* on isolated aortic rings.

## Material and methods

### Materials

Acetylcholine, nitroglycerin and sodium nitroprusside were from Sigma (Markham, ON, Canada). Angiotensin II and endothelin-1 peptides were acquired from Peninsula Laboratories Inc. (San Carlos, CA)

### Animals and Study Design

Twenty-four male New Zealand White rabbits (3–4 kg body weight) were used in this study. Animals were treated in accordance to the *Guide to the care and use of experimental animals* published by the Canadian Council on Animal Care and the protocol was approved by the Animal Protection Committee of the Université Laval. Sixteen rabbits were divided in two groups (n = 8) and all animals were fed with standard rabbit chow supplemented with 0.5% cholesterol (w/w) (Harlan, Indianapolis, IN) for 2 or 8 weeks respectively. The other 8 animals received normal rabbit chow for eight weeks (normal controls). After 2 weeks, 8 randomly chosen cholesterol-fed rabbits were killed; the others were kept alive for an additional 6 weeks (total of 8 weeks of hypercholesterolemic diet) as for the normal control group.

When animals were sacrificed abdominal and thoracic aortas were excised and immediately rinsed in freshly prepared Krebs buffer in preparation for the *ex vivo* experiments. Plasma samples were drawn from the marginal ear vein every week and plasma cholesterol levels were determined using a commercially available spectrophotometric assay kit (Roche Molecular Biochemicals, Saint-Laurent, Canada).

### In vivo evaluation of endothelial function by trans-abdominal ultrasound

**Figure 1:**
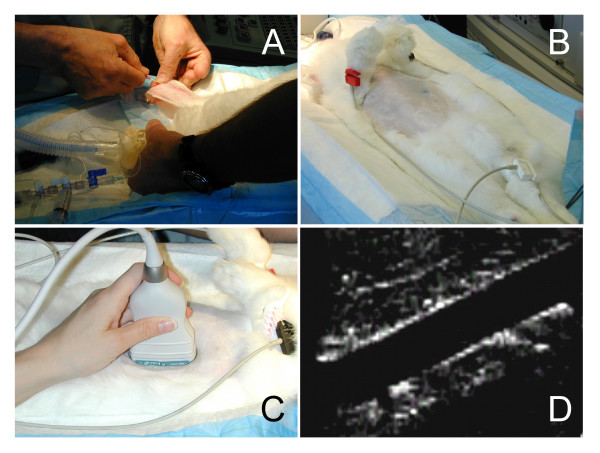
*In vivo* assessment of endothelial function in rabbits. Rabbits under sedation were serially infused for two minutes in the marginal ear (A) vein with the vehicle. An ink mark (Panel B) was made on the rabbit abdomen for probe location for each exam. Images (Panels C and D) of the abdominal aorta were recorded throughout the procedure.

Ultrasound evaluation of endothelial function of the abdominal aorta was performed at baseline, 2 weeks and 8 weeks. Rabbits were sedated using midazolam (0.5 mg/kg), butorphanol (0.5 mg/kg) and ketamine (30 mg/kg) IM. Marginal ear vein and artery were cannulated for drug infusions and arterial blood pressure monitoring, respectively (Figure 1A). Heart rate was monitored continuously throughout the procedure. The abdomen was shaved and the animal was put in dorsal decubitus (Figure 1B and 1C). The abdominal aorta was located using two-dimensional and color Doppler ultrasound. Image settings were optimized to allow the best and clearest definition of the endothelial-blood interface (Figure 1D). All studies were performed with a vascular 7.5 MHz probe coupled to a Sonos 5500 echograph (Phillips, Andover, MA).

**Figure 2:**
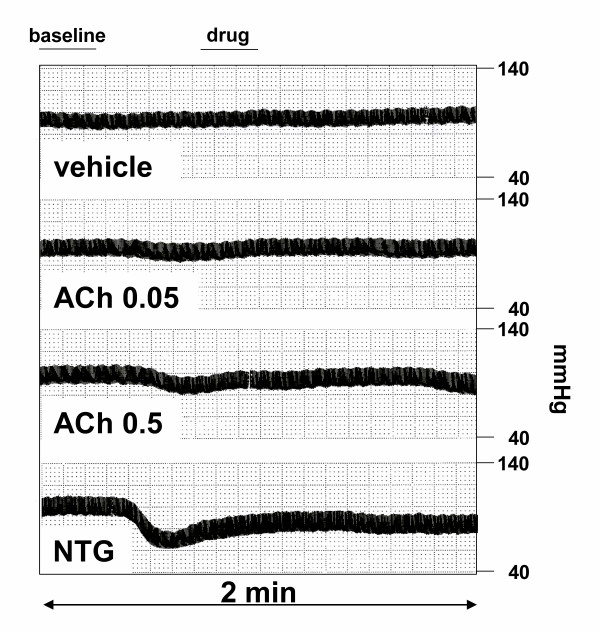
Representative blood pressure recordings during vehicle and drug infusions. Video sequences from the first 15 seconds (void volume) of drug infusion at baseline and between 40 to 60 seconds of drug infusion were digitized and used for analysis.

Once the imaging of the aorta was considered optimal, the animals received the following drug perfusions I.V sequentially for 2 minutes each: 1) saline at 1 ml/min; 2) acetylcholine (Ach) at 0.05 μg/ml/min and Ach at 0.5 μg/ml/min. Nitroglycerin (5 μg/ml/min) was used as positive control. Typical arterial blood pressure recordings are illustrated in Figure 2. At the end of a drug infusion, blood pressure was allowed to come back to baseline for at least one minute before the next infusion was started. Images of the abdominal aorta were recorded continuously through the entire procedure on standard S-VHS videotape for off-line analysis.

### Image analysis

Video sequences from the first 15 seconds (void volume) of drug infusion at baseline and between 40 to 60 seconds of drug infusion were digitized (Dazzle Video Creator, Dazzle Multimedia, Fremont CA) and stored on a computer for analysis (Figure 2). Still frames of the aorta from both the baseline and drug infusion (n = 5 each) synchronized to the beginning of the QRS and the respiratory phase were analyzed. The maximal diameter was measured (5 beats averaged) using the SigmaScan Pro software (SSSP Science, Chicago IL). Care was taken to measure the same segment for each beat using anatomic landmarks as a guide. The mean of 5 diameters for each image of the aorta was calculated. Vessel cross-sectional area was then calculated assuming the aortic section is circular using the formula: Area = π (D/2)^2^ where D is the diameter of the aorta. Area was expressed in percent of change from baseline. Inter and intra-observer variability was assessed on 10 randomly selected studies.

### Ex vivo endothelial function evaluation in isolated rabbit aortic rings

At the end of the protocol rabbits were given a sub-lethal dose pentobarbital (25 mg/kg) and were sacrificed by exsanguination. The middle part of the descending thoracic aorta as well as the abdominal aorta were removed and dissected free of adhering fat and connective tissues. The aorta was placed in warm Krebs solution. Rings of 5 mm thickness were suspended in individual organ chambers filled with 5 ml of oxygenated Krebs (37C pH 7.4). The segments were connected to force transducers and any variations in force were recorded continuously (WIN-SMT software, PO-NE-MAH inc., Gould, Valley View, OH.).

### Contractile response

Baseline contractile response was evaluated by a 30 to 60 minutes exposition to KCl (80 mM) where the rings were gradually stretched to a resting tension of 2 g until steady state was reached. Following this initial experiment, contractile capacity was further evaluated by exposing the rings to other vasoconstrictors. Briefly, when the rings had recovered their resting tension after the initial KCL exposure, they were exposed sequentially to cumulative concentrations of L-phenylephrine (PE, 10^-9^ to 10^-5^ M), angiotensin II (10^-10^ to 10^-7^ M) and endothelin-1 (10^-9^ to 10^-6^ M). Results were compared to the initial response obtained with KCL 80 mM.

### Relaxation response

Relaxation studies were performed after a precontraction with PE (10^-6^ M). Cumulative concentrations of acetylcholine (10^-9^ to 3 × 10^-6^ M) or sodium nitroprusside (10^-10^ to 3 × 10^-5^ M) were used. Sodium nitroprusside was used as a non-endothelial dependant vasodilator while acetylcholine evaluated the endothelial-dependant vasodilatation. Relaxation was expressed as a percent of change from the pre-contracted tension with PE.

### Statistical analysis

Results are expressed as mean ± standard error of the mean (SEM). Differences between the various conditions in the *in vivo* endothelial function experiments were evaluated with an ANOVA for repeated measures using the Tukey's post hoc test to evaluate significance. In the *in vitro* study, Student t-test was used when two values were compared. Differences were considered significant when p < 0.05.

## Results

### Total cholesterol circulating levels

**Figure 3:**
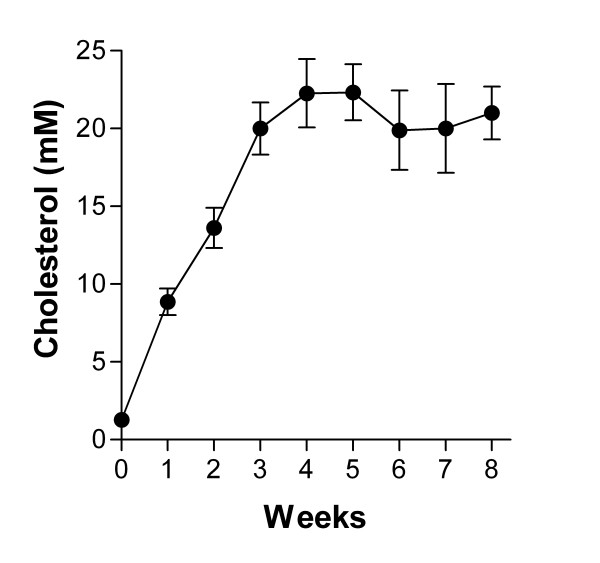
Total cholesterol circulatory levels in rabbits fed with the cholesterol diet. Results are expressed as mean ± SEM in mmoles/l (mM). (n = 8 animals)

Total cholesterol levels were measured weekly in the serum of cholesterol-fed rabbits. As illustrated in figure 3, total cholesterol levels rose sharply after one week of hypercholesterolemia and stabilized around 20 mM from Week 3 to Week 8.

### In vivo experiments

**Figure 4:**
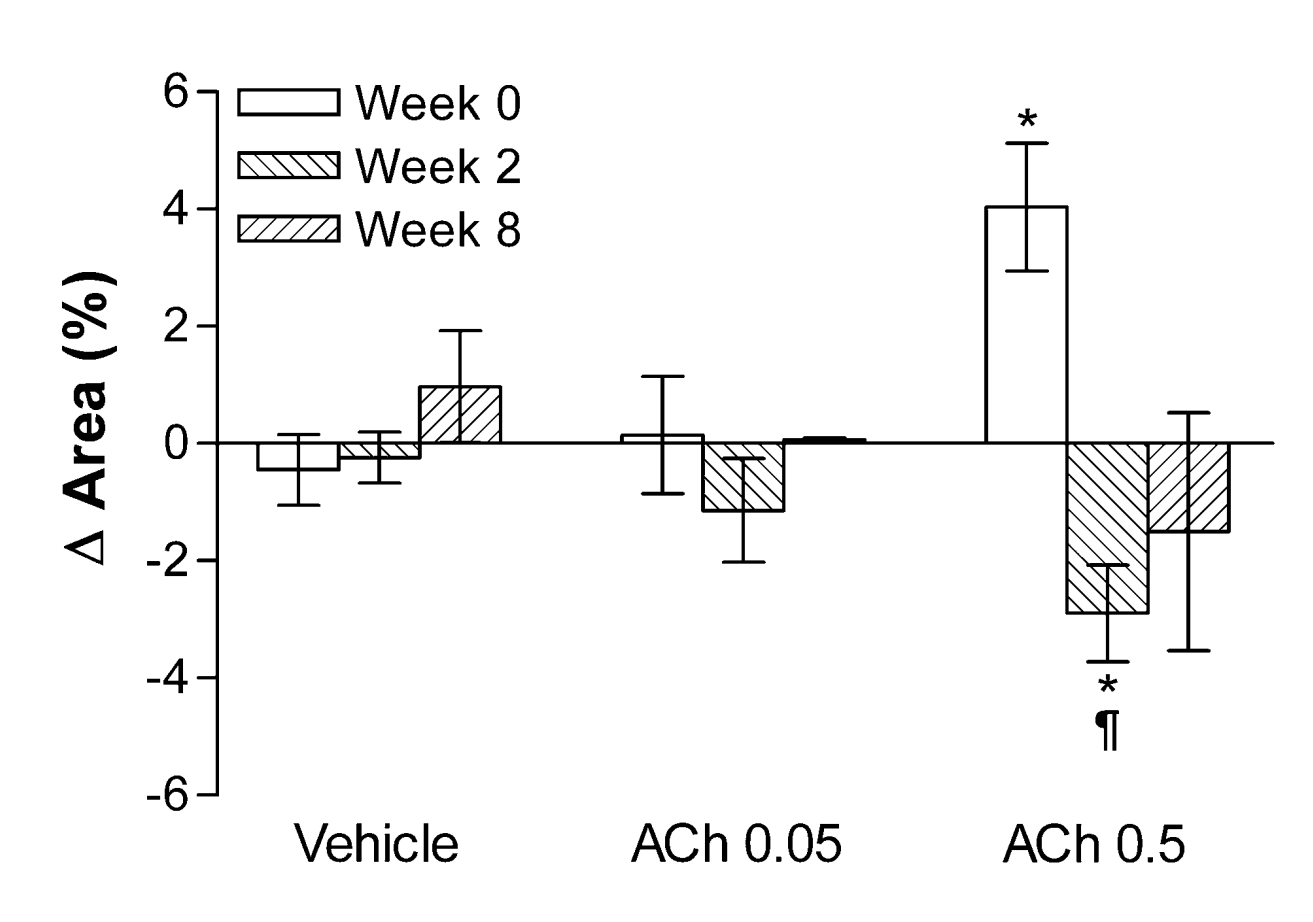
*In vivo* assessment of endothelial function in rabbits. Change in the area of the abdominal aorta in response to acetylcholine infusions. Rabbits (n = 8) were fed with a cholesterol diet for the indicated period of time. The endothelial function was assessed before (week 0) and after 2 (week 2) and 8 (week 8) of hypercholesterolemia. Rabbits under sedation were serially infused for two minutes (saline (vehicle); 1 ml/min) in the marginal ear vein with the vehicle (left), acetylcholine (Ach) at 0.05 and at 0.5 mg/ml/min. *: *P* < 0.05 vs. vehicle and ¶ *P* < 0.05 vs Week 0.

As expected, saline alone had no effect. Low doses of acetylcholine (ACh 0.05 and ACh 0.5 μg/kg/min) had only a minor and transient lowering effect on blood pressure. As illustrated in Figure 4, both saline and ACh 0.05 infusion had no effect on abdominal aortic cross-sectional area compared to baseline in both normal (Week 0) and hypercholesterolemic (Week 2 and 8) rabbits. As expected, ACh 0.5 infusion induced a dilatation of the aorta in animals at week 0 but had a paradoxical effect (contraction) at Week 2. Interestingly, while the response was clear and homogeneous at 2 weeks, it was more heterogeneous after eight weeks of hypercholesterolemia as endothelial function had improved in 5/8 rabbits (62%) and 2/8 (25%) regained a normal endothelial function. Although a trend towards contraction was recorded overall at week 8, it did not reach statistical significance.

### Ex vivo endothelial function measurements

**Figure 5:**
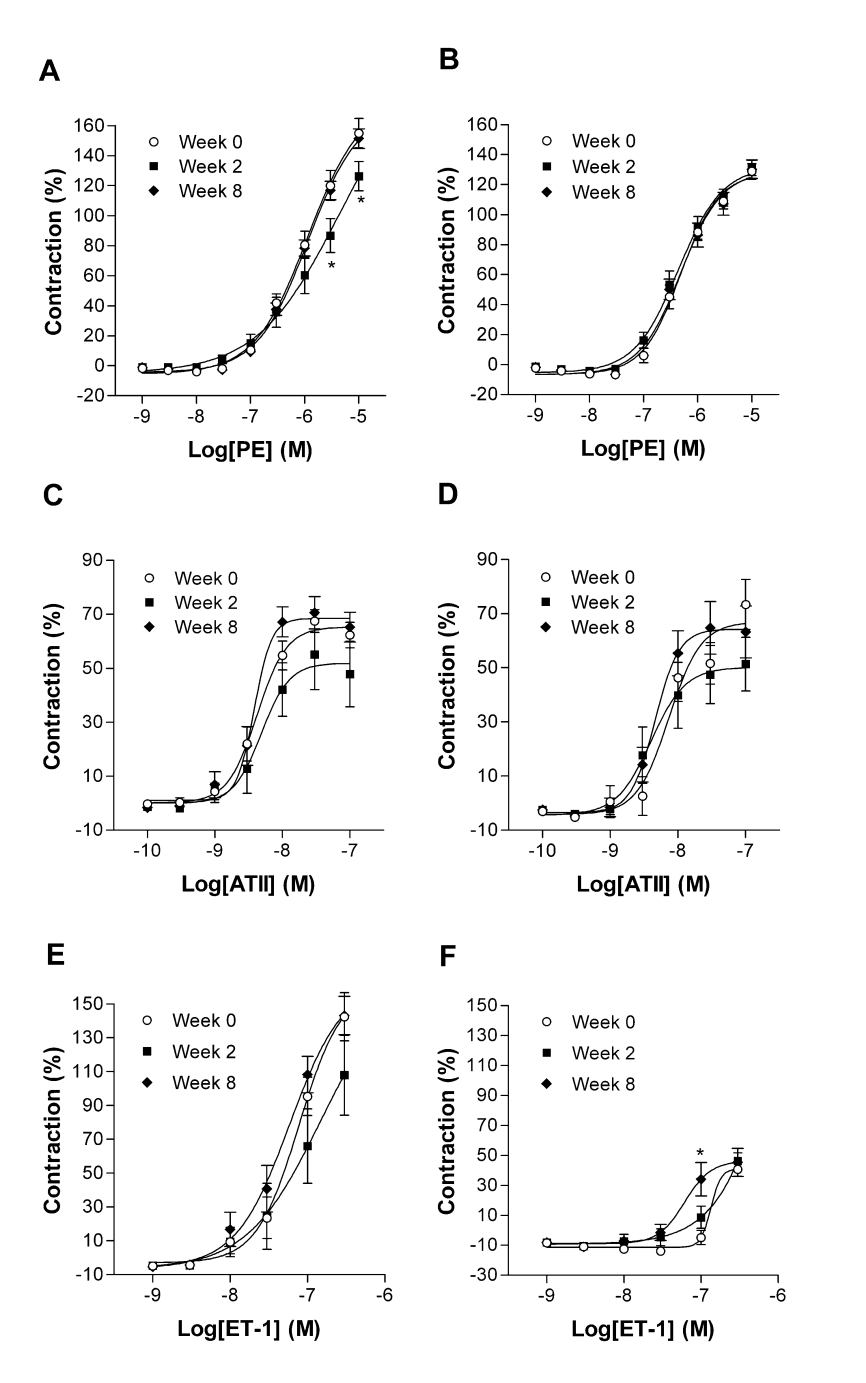
Contraction to phenylephrine (PE), angiotensin II (ATII) and to endothelin-1 (ET-1) of abdominal (panels A, C and E) and thoracic (panels B, D and F) aortic rings from rabbits fed or not with a cholesterol diet for 2 or 8 weeks. Aortic rings were exposed to cumulative doses of the indicated agent. Values are presented as percentage of contraction relative to a KCl (80 mM) control contraction. Values are expressed as mean ± SEM (n = 16). *P < 0.05 vs Week 0.

In order to confirm the validity of the *in vivo* results, we performed sections isometric contraction-relaxation experiments on isolated aortic rings. In Figure 5 are illustrated the contraction experiments using phenylephrine (Fig. 5A and 5B), angiotensin II (Fig. 5C and 5D) and endothelin-1 (Fig. 5E and 5F). All results are expressed relative to a control contraction using potassium chloride (80 mM). Except for endothelin-1, responses to vasoconstrictor were similar between abdominal and thoracic portions of the rabbit aorta. Thoracic aorta was less responsive to endothelin-1 than the abdominal portion. The amplitude of this response to endothelin-1 was also clearly less in the thoracic aorta.

**Figure 6:**
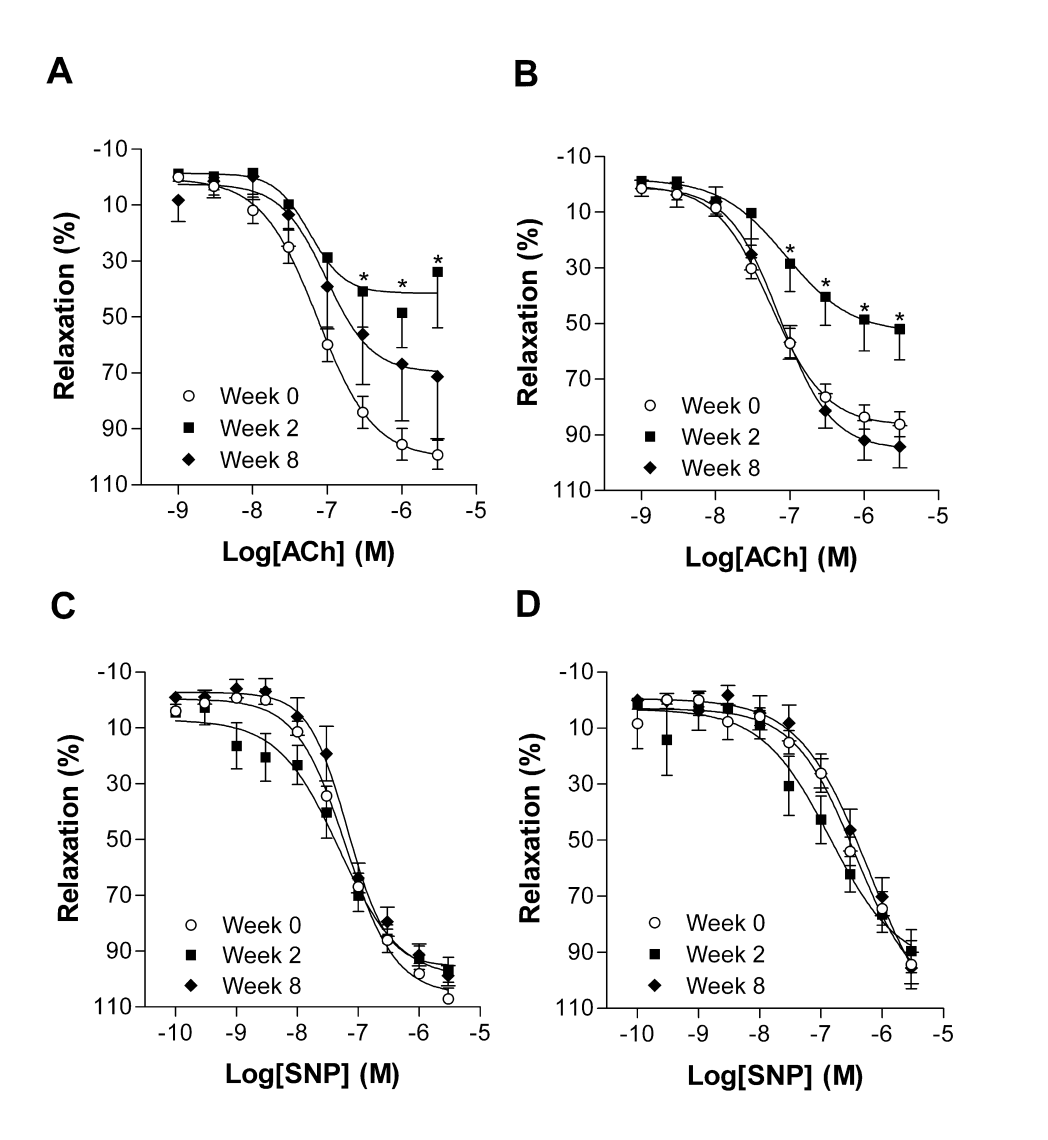
Relaxation to acetylcholine (ACh) and to sodium nitroprusside (SNP) of abdominal (panels A and C) and thoracic (panels B and D) aortic rings from rabbits fed or not with a cholesterol diet for 2 or 8 weeks Aortic rings were exposed to cumulative doses of the indicated agent. Values are presented as percentage of relaxation relative to a phenylephrine precontraction (1 μM). Values are expressed as mean ± SEM (n = 8). *P < 0.05 vs Week 0.

We then studied the endothelium-dependent relaxation using acetylcholine on our aortic rings after a pre-contraction with phenylephrine (1 μM). As illustrated in Figure 6A and 6B, abdominal and thoracic aortic ring of rabbits fed for two weeks with a cholesterol diet had a decreased vasodilatory response compared to normal rabbits (Week 0).

As seen *in vivo*, the endothelial function of the animals fed 8 weeks with the cholesterol diet was heterogeneous. In those animals hypercholesterolemia had no effect on the acetylcholine-induced relaxation of thoracic aortic rings while for the abdominal aortic sections; the response to acetylcholine was highly variable. As illustrated in Figure 6C and 6D the endothelium-independent response to sodium nitroprusside of aortic rings was normal and similar for all treatments and controls.

## Discussion

Our results clearly show that endothelial function can be assessed non-invasively by transcutaneous ultrasound of the abdominal aorta in hypercholesterolemic rabbits. The method was easily feasible in all animals and yielded very reproducible results. We also show that this in vivo method correlates very well with the ex vivo evaluation of endothelial function on isolated aortic rings. To our knowledge, this is the first demonstration of such a comparison.

Ultrasound imaging of the brachial artery in response to reactive hyperaemia has been used in many studies in humans [6-12]. Normal arteries dilate in response to reactive hyperaemia while arteries with an abnormal endothelial function show a decreased or absent response to this stimulus. Ultrasound evaluation of the aorta in rabbits has been performed in the past mostly to evaluate the extent of atherosclerotic plaques in response to a hypercholesterolemic diet. Intravascular ultrasound has been used to document endothelial dysfunction but has never been compared to *ex vivo* evaluation of endothelial function on aortic rings [1-5]. Our method is much less invasive for the animals than intravascular ultrasound. It is compatible with longitudinal studies requiring repeated measurements in the same animal and correlates very well with the *ex vivo* data [13,14].

The extent of endothelial dysfunction observed after 2 weeks of hypercholesterolemic diet was surprising although this parameter has not been studied very much after such a short exposure to hypercholesterolemia in rabbits [2]. The paradoxical contraction in response to acetylcholine signs the presence of endothelial dysfunction and this is confirmed in bath studies. However, the results obtained after 8 weeks were unexpected. Indeed, a significant number of animals had an improved endothelial function after 8 weeks of hypercholesterolemia compared to the two weeks data both *in vivo* and *ex vivo*. This transient improvement of endothelial function in the early phases of the atherosclerotic process has never been described before to our knowledge and the underlying mechanisms responsible for this paradoxical response need to be explored. This dysfunction may relate to an initial stress response of the aortic endothelium to hyperlipidemia then evolving with the development of atherosclerosis lesions.

## Conclusion

Endothelial function can be evaluated non-invasively in rabbits using a standard vascular ultrasound probe by a trans-abdominal approach. Results correlate well with *in vitro* data. A transient improvement in endothelial function can occur after 8 weeks of hypercholesterolemia in some animals for reasons that remain unclear.
